# Double Heterotopic Pregnancies with Live Intrauterine Pregnancy after Ovarian Stimulation

**DOI:** 10.1155/2022/7520243

**Published:** 2022-02-08

**Authors:** S. Shi, M. Rasouli, A. Raman, G. de Guzman

**Affiliations:** Department of Obstetrics and Gynecology, Kirk Kerkorian School of Medicine at UNLV, University of Nevada Las Vegas, 3016 W. Charleston Blvd., Suite 205, Las Vegas, NV 89102, USA

## Abstract

Heterotopic pregnancies, although rare in natural conceptions, have increased in incidence with use of assisted reproductive techniques (ART). Double heterotopic pregnancy in addition to an intrauterine pregnancy is exceedingly rare. In this case, we present a patient who underwent ovulation induction and intrauterine insemination (IUI) and was found on ultrasound to have a live heterotopic pregnancy. Intraoperatively, both fallopian tubes were grossly swollen and engorged. Bilateral salpingectomy was performed. Pathology identified gestational products in both fallopian tubes consistent with a double heterotopic pregnancy. Postoperatively, the intrauterine pregnancy resulted in a live birth. Although double heterotopic pregnancy and an intrauterine pregnancy is exceedingly rare, this case emphasizes the importance of routinely inspecting the contralateral fallopian tube at the time of surgery for an ectopic pregnancy, particularly in patients undergoing ovulation induction.

## 1. Introduction

Heterotopic pregnancy describes the presence of simultaneous pregnancies at different implantation sites. More often, these sites are a combination of an intrauterine with one or more extrauterine pregnancies. Heterotopic pregnancy is historically rare with a frequency of 1 in 30,000 spontaneous pregnancies [1]. More recent data estimates higher rates of 1 : 100–1 : 8000, with the highest rates occurring in patients undergoing assisted reproductive technologies (ART), such as ovulation induction, intrauterine insemination (IUI), and in vitro fertilization (IVF) [2, 3].

The diagnosis of heterotopic pregnancy can be challenging, as human chorionic gonadotropin (hCG) analysis may be within the normal range and ectopic pregnancies can be missed on ultrasound if an intrauterine gestation is seen and clinical suspicion for heterotopic pregnancy is low. We present a rare case of heterotopic pregnancy involving bilateral tubal pregnancies with simultaneous intrauterine pregnancy in a patient who underwent ovulation induction and IUI.

## 2. Case

The patient is a 36-year-old G2P0010 with polycystic ovarian syndrome. The patient presented with a history of oligomenorrhea and was found to have clinical hyperandrogenism on physical examination. She was undergoing fertility treatment at a private fertility clinic. Her infertility evaluation was significant for elevated total testosterone at 55, AMH of 3, and normal hysterosalpingogram demonstrating bilateral patent fallopian tubes and a normal semen analysis for her partner. The patient underwent ovulation induction with human recombinant follicle stimulating hormone (rFSH) and IUI. After IUI, the patient had a positive pregnancy test. At her follow-up visit, ultrasound at the fertility clinic confirmed a live intrauterine pregnancy at 6 weeks and 2 days of gestation without adnexal masses.

**Figure 1:**
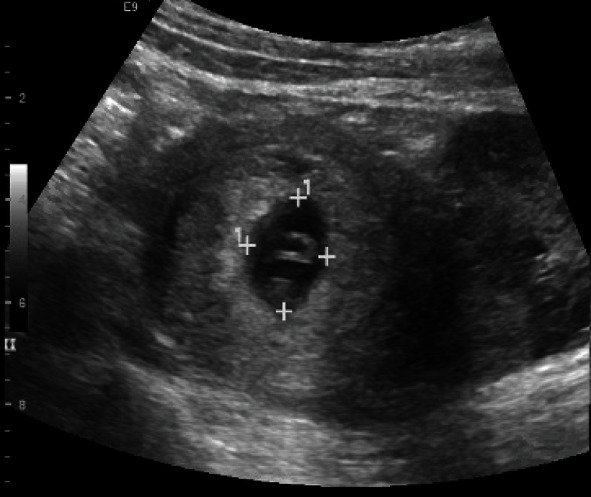
Intrauterine pregnancy with crown rump length 0.98 cm consistent with 7 weeks and 0 days of gestation.

**Figure 2:**
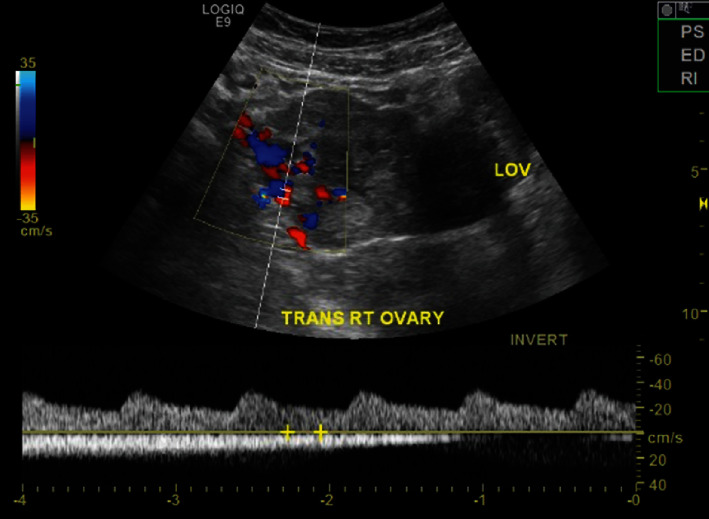
Right adnexa with Doppler flow.

**Figure 3:**
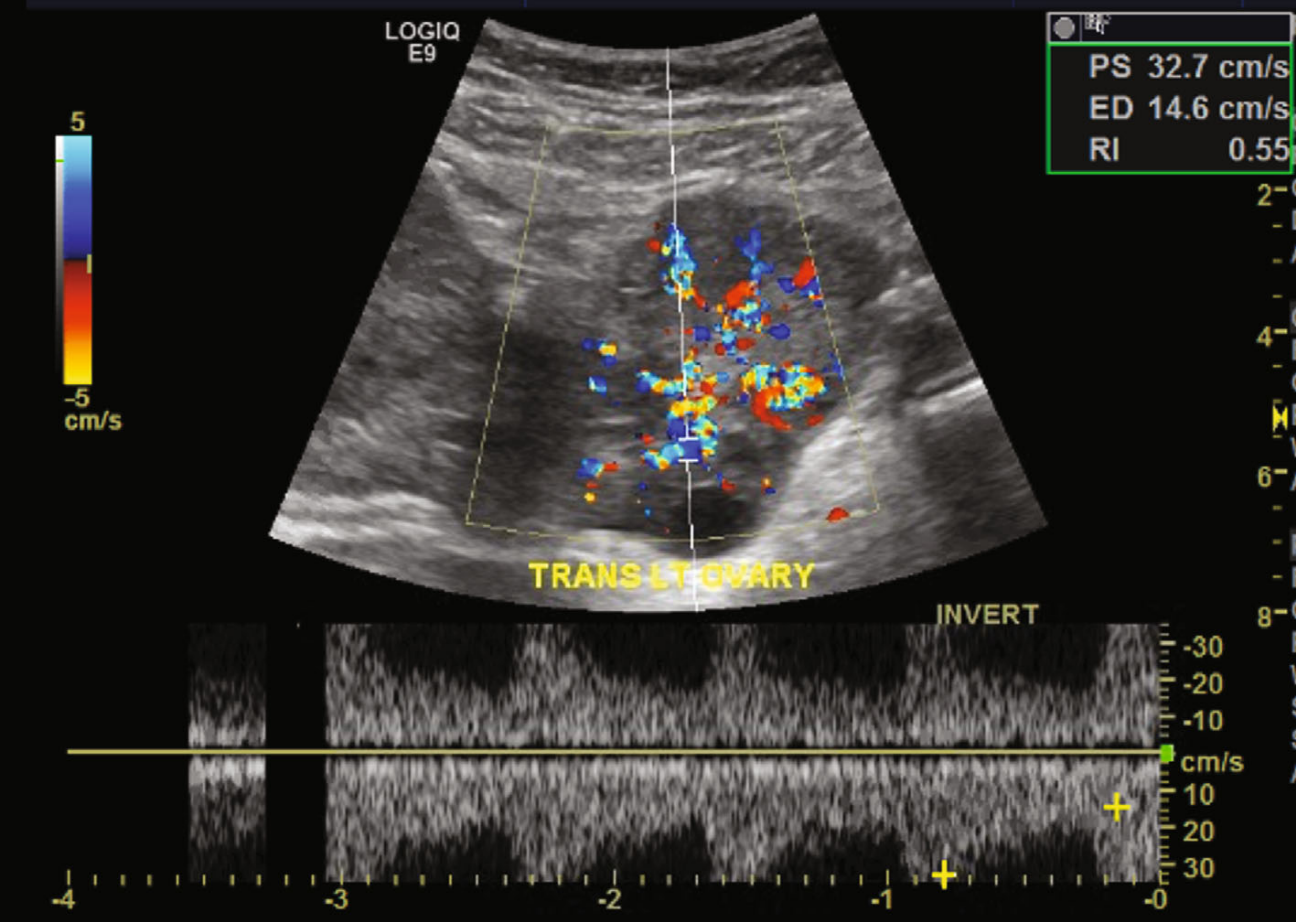
Left adnexa with Doppler flow.

**Figure 4:**
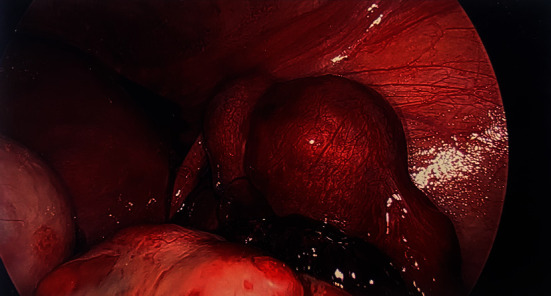
Intraoperative laparoscopic findings of bilateral tubal masses with gravid uterus.

She presented to the emergency room at 7 weeks and 1 day of gestation for evaluation of neck pain which was determined to be musculoskeletal in origin. She also endorsed about 2 weeks of intermittent vaginal spotting; however, she denied abdominal pain. On physical exam, the patient had a blood pressure of 119/69 mm Hg and a heart rate of 98 beats per minute. On physical exam, there was mild diffuse lower abdominal tenderness without rebound tenderness or guarding. Laboratory testing revealed a hemoglobin level of 11.4 mg/dL and a serum *β*-human chorionic gonadotropin level of 56,535 mIU/mL. Transvaginal pelvic ultrasound was done in the emergency room (Figures 1–3). This ultrasound demonstrated a live intrauterine pregnancy measuring at 7 weeks and 1 day of gestation, a live right ectopic pregnancy at 6 weeks and 5 days of gestation, and a complex left adnexal cyst. The patient was counseled about the ultrasound findings, and given this was a highly desired pregnancy, she was consented for diagnostic laparoscopy and possible salpingostomy or salpingectomy. Intraoperatively, the right fallopian tube was noted to be grossly swollen and discolored and had an area of concerning for rupture with adherent blood clot (Figure 4). A right salpingectomy was performed. Attention was then turned to the left fallopian tube, which was also noted to be swollen and engorged. The abnormal appearance of the left fallopian tube with the history of ovulation induction was highly concerning for a second ectopic pregnancy. Due to this finding, decision was made to perform a left salpingectomy in addition to the right salpingectomy. Both fallopian tubes were sent to pathology. The patient tolerated the procedure well and was discharged home the same day of surgery. The pathology report was notable for gestational products in both fallopian tubes indicating that she had two pregnancies in the fallopian tubes and one intrauterine pregnancy. The patient was seen in clinic for follow-up on postoperative day 8, and a live intrauterine pregnancy was reconfirmed at that time. The patient went on to have an uncomplicated pregnancy resulting in a vaginal delivery of a healthy, full-term infant. She gave written consent for publication of this report.

## 3. Discussion

Bilateral tubal pregnancies and heterotopic pregnancies are rare in spontaneous conceptions, but their incidence has risen significantly with the advent of assisted reproductive techniques including IUI, superovulation, and IVF [4]. The incidence of heterotopic pregnancies with assisted reproductive techniques is estimated to be about 30 to 60 times higher as compared to natural conceptions [5]. The diagnosis of ectopic and heterotopic pregnancy should always be considered in the differential for these patients as a delayed diagnosis can lead to undesirable and life-threatening complications. However, diagnosis of heterotopic pregnancy can be challenging, especially at early gestation as presence of an intrauterine pregnancy could mask the need for complete evaluation of adnexa in an asymptomatic patient and hCG evaluation can be falsely reassuring. We report a rare case of heterotopic pregnancy including bilateral tubal ectopic pregnancies with simultaneous intrauterine pregnancy that was conceived by ovulation induction with rFSH and IUI. There should be a heightened awareness of possible heterotopic pregnancy in patients that have undergone ART treatment. Performing high-resolution transvaginal ultrasound with close scanning of adnexa can help in early diagnosis and potentially prevent life-threatening complications [6, 7]. In the effort to have better outcomes for intrauterine pregnancy and minimize harmful effects to the intrauterine gestation, management of heterotopic pregnancy should be approached in a minimally invasive approach and typically involves laparoscopy with either salpingostomy or salpingectomy [8, 9]. Studies have shown that with early diagnosis and treatment, 63-70% of intrauterine pregnancies will reach viability [10–12]. This case also demonstrates a dilemma in management of tubal pathology noted at time of laparoscopy in patients desiring future fertility. In the case above, although the right fallopian tube was demonstrated on imaging to have a live ectopic, the left fallopian tube was also unexpectedly found to be grossly abnormal at the time of surgery. In this case, this was a highly desired pregnancy, and in the effort to prevent putting the patient at risk of a life-threatening event by leaving an ectopic pregnancy, the decision was made to perform bilateral salpingectomy. Although double heterotopic pregnancy in a patient with a live intrauterine pregnancy is an extremely rare but potentially catastrophic occurrence, to avoid unwanted complications from a delayed diagnosis, it is recommended to routinely inspect the contralateral fallopian tube at the time of surgery for an ectopic pregnancy, particularly in patients that have received ART treatment. Perhaps implementation of a checklist for early pregnancy verification, particularly in women who conceived by ART, could be an opportunity to implement a systems change to help improve sonographic implementation of such cases.
